# Attitude and perception toward artificial intelligence among German physicians with intensive care experience: a survey study

**DOI:** 10.3389/frhs.2025.1721620

**Published:** 2026-02-05

**Authors:** G. D. Giebel, P. Raszke, M. Tokic, L. Palmowski, N. Timmesfeld, H. Nowak, M. Adamzik, P. Heinz, S. Mreyen, F. M. Brunkhorst, J. Wasem, F. Buchner, N. Blase

**Affiliations:** 1 Institute for Health Care Management and Research, University of Duisburg-Essen, Essen, Germany; 2 Department of Medical Informatics, Biometry and Epidemiology, Ruhr University Bochum, Bochum, Germany; 3 Department of Anesthesiology, Intensive Care Medicine and Pain Therapy, Knappschaft Kliniken University Hospital Bochum, Ruhr-University Bochum, Bochum, Germany; 4 Department of Anesthesiology, Intensive Care Medicine and Pain Therapy, Center for Artificial Intelligence, Medical Informatics and Data Science, Knappschaft Kliniken University Hospital Bochum, Ruhr-University Bochum, Bochum, Germany; 5 Knappschaft Kliniken GmbH, Recklinghausen, Germany; 6 Institute of Infectious Diseases and Infection Control, Jena University Hospital, Jena, Germany; 7 German Sepsis Society, Berlin, Germany; 8 Chair of Health Economics, Kärnten University of Applied Sciences, Feldkirchen, Austria

**Keywords:** artificial intelligence—AI, digital health, intensive care medicine, medical decision-making, physician attitudes, survey study

## Abstract

**Introduction:**

The applications of artificial intelligence (AI) in healthcare are very diverse. AI-based systems can assist with diagnosis and decision-making, particularly in intensive care medicine. However, physicians must accept these systems to fully exploit their potential. We investigated attitude and perception toward AI among physicians with intensive care experience.

**Methods:**

A cross-sectional questionnaire survey was conducted between August and October 2024 among 7,475 physicians with intensive care experience. Participants were recruited via the hospital operator Knappschaftskliniken GmbH, the German Sepsis Society and via an address register. The questionnaire collected background information on the participants as well as their attitude toward and perception to AI. Their general attitudes toward AI were assessed using the validated Attari-12 tool. Questions specifically addressing attitude and perception of AI in healthcare were developed independently. Descriptive statistics and subgroup analysis were conducted.

**Results:**

Of the 7,475 physicians initially contacted, 620 returned the questionnaire. Of these, 445 questionnaires were included in the evaluation. Most were male (81.8%) aged over 50 years in leadership positions (92.1%). In both cases, general and health care specific, the attitude toward AI was rather positive. The majority of physicians asked for AI applications that are comprehensible to the treating physicians (87.1%) and agreed that objective values alone are not always sufficient for making medical decisions (87.3%). Furthermore, physicians faced problems in finding reliable information about AI in healthcare (52.6%) and only 21.6% considered communication about AI in the medical community as appropriate. Subgroup analysis revealed few differences for age and gender. The correlation between conscious use of AI in a professional context and attitude toward it was notable.

**Discussion:**

Physicians with intensive care experience generally hold a positive attitude toward AI, particularly in healthcare. However, the sample was predominantly male, older, and in leadership positions, so these findings may not fully reflect the attitudes of younger or female physicians. Several considerations were highlighted: AI outputs should be interpretable, clinical decisions cannot rely solely on objective data, and physicians need reliable information and guidance for further AI education. Leveraging the positive attitude could help make healthcare systems more efficient, effective, and sustainable.

## 1 Introduction

The idea of using Artificial Intelligence (AI) in medicine is not particularly new. Back in the early 1970s, the AI-based expert system MYCIN was developed to identify different bacteria and to recommend suitable antibiotics (1). Nevertheless, today, more than 50 years later, the adoption of AI in medicine remains uneven, with some specialties using it more extensively than others. This is surprising, given the wide range of possible applications. These include drug development, health monitoring, managing medical data, disease diagnostics, digital consultation, personalized treatment, analysis of health plans, surgical treatment, and medical treatment (2).

In August 2025, the FDA AI/ML-Enabled Medical Devices database listed 1,247 devices using AI. Thereof 956 (76.7%) stem from the field of “Radiology”, 116 (9.3%) systems are listed under the category “Cardiovascular”, 56 (4.5%) under “Neurology”, 22 (1.8%) under “Anesthesiology”, 19 (1.5%) under “Hematology”, 17 (1.4%) under “Gastroenterology-Urology”, and 10 (0.8%) under “Ophthalmic” (3). These numbers indicate a low occurrence of such systems in intensive care. However, this contrasts with the wide range of potential application of AI in the respiratory, renal and cardiovascular systems, as well as in trauma, delirium and sepsis (4). Especially, the use of AI in the field of sepsis is promising. AI-based clinical decision support systems (CDSS) can reliably predict the occurrence of sepsis or septic shock and can help to individualize sepsis therapy to reduce mortality (5).

In order to conduct further research into the use of AI-based CDSS in sepsis care, the KI@work research project was initiated in 2022 (6). The objective of this project is twofold: first, to identify problems and the barriers that impede the deployment of such systems; and second, to elicit intensive care physicians’ requirements for AI-based CDSS, using sepsis as an example. In accordance with the first objective, expert interviews were conducted (7, 8). The analysis of these interviews indicated that user perception of AI was frequently described as problematic. This finding is of particular concern, since prior work has shown that perception and need are closely linked to the adoption of AI in healthcare (9). Should physicians hold unfavorable perceptions of AI, this could therefore pose a significant barrier to its acceptance and effective use.

Established theoretical frameworks also emphasize that the way a technology is perceived strongly influences its adoption. Thus, according to the Technology Acceptance Model (10), perception is an essential influence shaping attitude toward new technologies. Similarly, the Unified Theory of Acceptance and Use of Technology (UTAUT) stresses the importance of perception and attitude (11), with constructs such as “Performance Expectancy”, “Effort Expectancy”, “Social influence” and “Facilitating Conditions” preceding “Behavioral Intention”. Building on these frameworks, Lambert et al. reviewed the acceptance of artificial intelligence among healthcare professionals in hospitals (12). To do so, they collected studies with real AI applications. Hua et al. pursued a related approach, investigating factors influencing the acceptability of AI in medical imaging domains (13). While most of the studies included in the reviews aimed on specific systems, those with a more general focus on AI did not focus on intensive care.

Against this background, the present study aims to explore the fundamental attitudes of physicians with intensive care experience toward AI in general and especially in healthcare.

## 2 Methods

A cross-sectional survey was conducted among German physicians with intensive care experience. The reporting of the study follows the “Consensus-Based Checklist for Reporting of Survey Studies” (CROSS) (14). This approach is intended to ensure transparency and comprehensiveness in reporting.

### 2.1 Data collection methods

A questionnaire was designed based on theoretical groundwork in the form of expert interviews (7, 8). It consists of four parts (Table 1). An excerpt of the questionnaire depicting the relevant questions is provided in Supplementary Appendix 1. The results of the discrete choice experiment (DCE) in part three are not included in the results presented here. Nevertheless, the DCE required the use of five versions of the questionnaire in order to vary the choice sets. However, this did not affect the components examined in this article, as the structure, number and sequence of all other questions were identical across versions. As this article focusses on the attitude toward AI, as well as attitude and perception toward its use in healthcare, parts 1, 2.2 and 4 were evaluated (cf. Table 1).

**Table 1:** Structure of the questionnaire.

Part	Number of questions
1. Details of the person completing the form (including socio-demography) and their working environment	8 questions
2. Attitudes and perspectives:	34 questions
2.1 Clinical Decision Support Systems	11 questions
2.2 Artificial Intelligence (AI) in healthcare	7 questions
2.3 AI-based Clinical Decision Support Systems	16 questions
3. Discrete Choice Experiment (DCE) on the optimal design of AI-based Clinical Decision Support Systems	9 questions
4. Attitude toward AI (ATTARI-12)	12 questions

The first section of the questionnaire comprises questions relating to socio-demographic characteristics (age and gender), medical specialization, specialist status, experience in intensive care, and whether the physician holds a leading position. With regard to the working environment, the estimated digital maturity of the hospitals was requested (1 = very good, 6 = insufficient). The second part of the study (2.2) investigates physicians’ attitude and perception toward AI in healthcare. The fourth part consists of the validated ATTARI-12 questionnaire (15). The latter consists of 12 questions designed to assess the general attitude toward AI. Half of the items are formulated in a positive, half are formulated in a negative way. In addition, four items each focus on the cognitive, affective and behavioral facet. After re-coding the negative formulated items (mirroring the scale), the mean value of all 12 items (providing the total general attitude toward AI) can be calculated. Both parts 2.2 and 4 use five-point Likert scales as response options (1 = strongly disagree, 5 = strongly agree).

### 2.2 Pretesting

A two staged pretest was conducted. The first stage included a think-aloud-protocol with four medical students (two female, two male). After revision, in stage 2, four experienced physicians with intensive care experience (all male) were asked to give verbal feedback. During the pretest, parts 1 and 2.2 were slightly improved for comprehensibility but not for content. The validated ATTARI-12 questionnaire in part 4 proved to be perfectly understandable. None of the pre-testers were included in the study sample.

### 2.3 Sample characteristics

The survey targeted physicians with intensive care experience (ward physicians, senior physicians and chief physicians). The recruitment process was predominantly facilitated through contact data provided by the Knappschaft Kliniken GmbH and the German Sepsis Society (DSG). In order to ensure an adequate sample size, additional physicians were contacted via purchased addresses from a private address provider. The provider applied a predefined selection algorithm determined by the project consortium: first, all available addresses of anesthesiologists and intensive care physicians were included, as these specialties routinely possess intensive care experience. The pool was then expanded to include orthopedics and trauma surgeons, general surgeons, and internists as these specialties are required to acquire intensive care experience during their residence training. For this second group, ward physicians were excluded, as they may not have sufficient intensive care experience required for the study. To avoid duplicates, entries already included in the DSG dataset or corresponding to hospitals of the Knappschaft Kliniken GmbH were removed. The physicians identified were contacted either by e-mail (Knappschaft Kliniken GmbH, *n* = 1,218), or by letter (DSG, *n* = 421; address provider, *n* = 6,950).

Participants were characterized by sex, age, specialist title, years of intensive care experience, additional qualification in intensive care medicine and leadership position. A comparison with the overall population of German physicians was conducted, and the results are presented in the Discussion (limitations section).

An *a priori* sample size calculation was performed to ensure sufficient power to detect group differences in the primary outcome. Assuming a two-tailed test, a normal distribution, an effect size of Cohen's d = 0.5, a significance level of *α* = 0.05, a power of 0.9, and equal group sizes, the required total sample size was estimated to be 180 participants. However, because the DCE part of the survey required a larger sample, this value (*n* = 472) was used as the target sample size for the study. Predefined exclusion criteria, listed in Textbox 1, were subsequently evaluated through the survey (cf. Supplementary Appendix 1).

Textbox 1 **Exclusion criteria.**

- No intensive care experiences
- Physicians with focus on pediatrics and adolescent medicine
- Physicians that cannot be categorized in one of the following groups:
  ○ Group 1: Surgically active physicians
  ○ Group 2: Acute and emergency medicine
  ○ Group 3: Physicians treating conservatively
- Attari-12 not fully completed

### 2.4 Survey administration

The medical practitioners contacted via post received a cover letter that provided information regarding the research project, data protection, and a paper questionnaire. This was accompanied by a stamped return envelope. The cover letter also included a Link and QR code that enabled respondents to complete the questionnaire online. The physicians who were contacted via e-mail received a digital cover letter containing the same information, as well as direct access to the questionnaire via link and QR code. The online questionnaire was implemented using Limesurvey (LimeSurvey GmbH). The dispatch of the questionnaires was executed in several steps between August and October 2024.

The questionnaires returned by post were digitalized by student assistants using the Scanner-Software Remark Office OMR (Gravic, Inc.). Inconclusive cases identified by the program were reviewed and corrected by hand. The digitalized data was saved in tables using Microsoft Excel (Microsoft). The questionnaires filled out online were downloaded as Excel files directly from Limesurvey. There was no IP tracking to prevent participants from completing the survey multiple times. The online questionnaire allowed participants to skip individual questions.

### 2.5 Ethical considerations

The survey was conducted on a voluntary basis and its objective was to investigate impersonal topics exclusively from health care providers aged over 18 years. Therefore, the Ethics Committee of the Medical Faculty of the University of Duisburg-Essen confirmed that an Ethics vote was not required. The final questionnaire was reviewed and endorsed by the works council (the employee representative body) of the Knappschaft Kliniken GmbH.

### 2.6 Statistical analysis

Data analysis was conducted with Python using Visual Studio Code (Microsoft). Descriptive statistics comprised frequency, percentage, mean, standard deviation, median, and range. Inferential tests were selected according to the measurement scale of the variables and the assumptions underlying each test. For binary independent variables, the Mann–Whitney *U* test was applied due to the ordinal nature of variables and potential violations of normality, except for the mean Attari-12 score. The latter was treated as approximately interval-scaled and therefore analyzed with the t-test. For independent variables with more than two ordinal categories, group differences were assessed using the Kruskal–Wallis test followed by *post-hoc* Dunn tests with Bonferroni adjustment, given that the assumptions of ANOVA (normality and homogeneity of variances) were not met.

To investigate the strength of the relationship between the dependent and independent variables, different association and correlation measures were calculated depending on the scale of the variables. For two-group comparisons with an ordinal dependent variable the effect sizes were calculated as *r*^2^ (*r*^2^ = Z^2^/N), where Z is the standardized test statistic (from the Mann–Whitney *U* test) and N is the total number of observations across both groups. This indicates how much of the variance in the dependent variable can be attributed to the different group affiliations. For associations involving more than two ordinal groups with an ordinal dependent variable Kendall's tau-c was calculated as an effect size to indicate the strength and direction of the trend across the ordinal groups. As the Attari-12 mean score was treated as approximately metric effect sizes were reported as Cohen's d. In all cases, *p*-values less than 0.05 were considered statistically significant.

Data sets with missing values among the Attari-12 questions were excluded from the analysis of general attitude toward AI. This approach may reduce statistical power and could introduce bias if the missingness was not completely random. Potential systematic non-response bias was not assessed or controlled during the survey. As a result, certain groups may be underrepresented, which could limit the generalizability of the findings. Neither sensitivity analysis nor weighting of the data was performed to adjust for these potential imbalances. A comparison was made with the overall population and is summarized in the discussion of the article.

## 3 Results

### 3.1 Respondent characteristics

The total number of individuals contacted was 7,475. This comprised 1,218 physicians who were employed by Knappschaftskliniken GmbH, as well as a further 6,257 physicians either from the German Sepsis Society or from the address register. A further 1,114 letters that were dispatched initially were not successfully delivered. 620 questionnaires were returned. This corresponds to a response rate of 8.3%. Following the implementation of the exclusion criteria (cf. Textbox 1), a total of 445 questionnaires (71.8%) were deemed eligible for inclusion in the present analysis.

The majority were male (81.8%) and older than 50 years (79.1%). 92.1% of participants stated to have a leadership position (senior physician or chief physician) and 98.2% had a specialist title. A minority (17.5%) had less than one year of intensive care experience. 37.3% had an additional designation in intensive care medicine. The characteristics of the respondents are listed in Table 2.

**Table 2:** Characteristics of respondents.

Characteristics	Participants, *n* (%)
Total	445
Gender	
Male	364 (81.8%)
Female	75 (16.9%)
n.a.	6 (1.3%)
Age (years)	
<41	30 (6.7%)
41–50	63 (14.2%)
51–60	187 (42.0%)
>60	165 (37.1%)
n.a.	0
Specialist title	
Yes	437 (98.2%)
No	6 (1.3%)
n.a.	2 (0.4%)
Intensive care experience (years)	
<1	78 (17.5%)
1–5	106 (23.8%)
6–10	36 (8.1%)
> 10	225 (50.6%)
n.a.	0
Additional designation: intensive care medicine	
Yes	166 (37.3%)
No	278 (62.5%)
n.a.	1 (0.2%)
Leadership position	
Yes	410 (92.1%)
No	32 (7.2%)
n.a.	3 (0.7%)
AI (knowingly) already used	
Yes	102 (22.9%)
No	327 (73.5%)
n.a.	16 (3.6%)

The participants evaluated the degree of digitalization in their hospitals as follows: very good (2.2%), good (18%), satisfactory (21.1%), sufficient (22%), poor (29.2%) and insufficient (6.5%). 44.7% felt they belonged to acute and intensive care medicine, 38.2% to surgical medicine, and 38.2% to conservative medicine.

### 3.2 General attitude toward AI

First, the Attari-12 tool was evaluated on an item-by-item basis. The analysis showed that participants tended to agree with positive statements and disagree with negative ones (cf. Table 3; Supplementary Appendix 2). The items that received the highest levels of agreement were ‘I want to use technologies that rely on AI’ (69.7% rather or totally agreed) and ‘I look forward to future AI developments’ (69% rather or totally agreed). The lowest levels of agreement were observed for the statements ‘I am afraid of AI’ (71.2% rather or totally disagreed) and ‘I would rather avoid technologies that are based on AI’ (78.2% rather or totally disagreed).

**Table 3:** Descriptive statistics of the attari-12 tool.

Item	Description	Mean	Standard deviation	Median	Range
1	AI will make this world a better place.	3.27	±0.99	3	1–5
2	I have strong negative emotions about AI.	2.31	±1.10	2	1–5
3	I want to use technologies that rely on AI.	3.85	±0.92	4	1–5
4	AI has more disadvantages than advantages.	2.53	±1.02	2	1–5
5	I look forward to future AI developments.	3.82	±0.99	4	1–5
6	AI offers solutions to many world problems.	3.38	±1.04	3	1–5
7	I prefer technologies that do not feature AI.	2.29	±1.13	2	1–5
8	I am afraid of AI.	1.99	±1.09	2	1–5
9	I would rather choose a technology with AI than one without it.	3.17	±1.01	3	1–5
10	AI creates problems rather than solving them.	2.35	±0.96	2	1–5
11	When I think about AI, I have mostly positive feelings.	3.05	±1.03	3	1–5
12	I would rather avoid technologies that are based on AI.	1.78	±0.97	1	1–5

**Figure 1:**
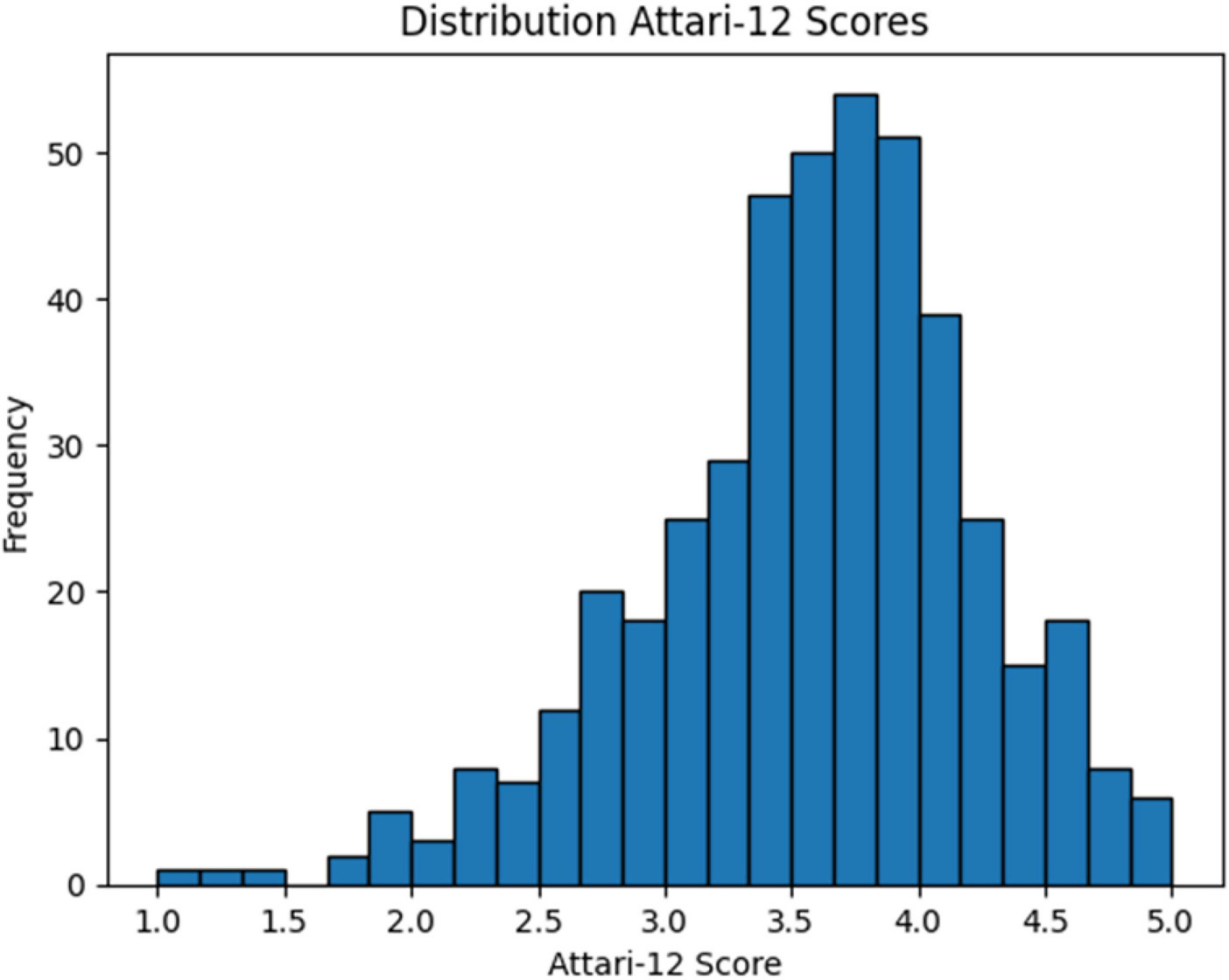
Histogram showing the distribution of the total attari-12 score.

After initial analysis of individual items, the negatively worded items were recoded, before taking the average of the 12 items, to calculate the general attitude toward AI (total Attari-12 score). The final score was 3.61 (±0.72) showing a generally positive attitude toward AI. The distribution is shown in Figure 1.

Following the descriptive analysis, subgroup analyses were conducted investigating the influence of gender, age and previous use of AI in professional context. Regarding gender, no statistically significant difference was found for the overall Attari-12 mean score [t(443) = −1.79, *p* = 0.07]. However, when examining the individual items, one statistically significant difference emerged for the eighth item ‘I am afraid of AI’ (U = 15,671, Z = 2.02, *p* = 0.03). In this case, though, the effect was small, with an explained variance of only *r*^2^ of 0.01.

For certain items subgroup analysis revealed statistically significant differences between individual age groups. However, correlations between age and the individual items (measured using Kendall's tau-c) were very weak and not statistically significant, indicating that these differences reflect only a minimal association. Regarding the mean Attari-12 score, no statistically significant differences were found between the age groups [H(3) = 6.05, *p* = 0.11]. The detailed subgroup analysis values can be found in Supplementary Appendix 3.

The subgroup analysis revealed the biggest differences among physicians who had or had not previously used AI professionally. This became especially apparent for total attitude, as measured by the Attari-12 mean. A significantly more positive attitude was observed in physicians with previous experience of using AI [t(443) = −2.99, *p* < 0.01]. The effect size was small to medium (Cohen's d = −0.324). Focusing on individual items, eight showed statistically significant differences in relation to previous professional use. These were ‘AI will make this world a better place.’ (U = 14,006, Z = −2.44, *p* = 0.01), ‘I have strong negative emotions about AI.’ (U = 19,923, Z = 2.97, *p* < 0.01), ‘I want to use technologies that rely on AI.’ (U = 13,247, Z = −3.14, *p* < 0.01), ‘I look forward to future AI developments.’ (U = 14,304, Z = −2.17, *p* = 0.02), ‘I prefer technologies that do not feature AI.’ (U = 19,919.5, Z = 2.97, *p* < 0.01), ‘I am afraid of AI.’ (U = 20,468, Z = 3.47, *p* < 0.01), ‘I would rather choose a technology with AI than one without it.’ (U = 14,413.5, Z = −2.07, *p* = 0.03), ‘When I think about AI, I have mostly positive feelings.’ (U = 14,222, Z = −2.25, *p* = 0.02). Even though the differences between groups reached statistical significance, the effect size (*r*^2^) showed that group membership had only a weak influence on attitudes (cf. Supplementary Appendix 3).

Further subgroup analyses regarding intensive care experience (in years), additional designation in intensive care medicine and leader ship position can be found in Supplementary Appendix 4. Observed effects were negligible, rarely reaching statistical significance, and if significant, only showed very small effect sizes.

### 3.3 Attitude and perception toward AI in the context of healthcare

While the Attari-12 questionnaire focusses on general attitude toward AI, this section specifically addresses its application in healthcare. Overall, physicians expressed a predominantly positive view. 80.6% reported a positive or completely positive attitude toward the use of AI in a medical context. 23.1% rather agreed and 64% totally agreed that results of AI must always be comprehensible to the treating physician. Similarly, 33.6% rather agreed and 53.7% totally agreed that objective values alone are insufficient for making medical decisions.

Regarding potential risks, physicians rarely had a clear opinion: only 4.1% totally agreed and 7% totally disagreed that using AI in medicine poses a risk to patients. In terms of accessing information, 31.2% rather disagreed and 21.4% totally disagreed about knowing where to find reliable information on AI in healthcare. Finally, only 3.8% totally agreed that communication about AI within the medical community was appropriate. Further descriptive statistics can be found in Table 4; Supplementary Appendix 5.

**Table 4:** Descriptive statistics on questions about AI in healthcare.

Description	*n*	Mean	Standard deviation	Median	Range
I am positive about the use of AI in a medical context	442	3.97	±0.93	4	1-5
I see a risk for patients when AI is used in a medical context	441	2.80	±0.98	3	1-5
The results of AI applications must always be comprehensible to the treating physician	442	4.25	±0.94	5	1-5
Objective values are not always sufficient for making medical decisions	441	4.36	±0.85	5	1-5
I know where to find reliable information (e.g., on evidence, general use) about AI in healthcare	426	2.60	±1.23	2	1-5
I consider communication about AI in the medical community (e.g., professional associations, conferences) to be appropriate	422	2.72	±0.99	3	1-5

Investigating differences between male and female physicians significancy was only identified for the item ‘I know where to find reliable information (e.g., on evidence, general use) about AI in healthcare’. Here, men significantly agreed more than women (U = 9,855.5, Z = −2.85, *p* < 0.01). However, the effect was low (*r*^2^ = 0.02). Subgroup analysis investigating on age revealed a statistically significant difference for the item ‘I know where to find reliable information (e.g., on evidence, general use) about AI in healthcare’ [H(3) = 12.06, *p* < 0.01]. However, there was no distinct correlation between age and the item (Kendall's τc = 0.069, *p* < 0.01). Finally, physicians with previous professional use of AI significantly agreed more to ‘I am positive about the use of AI in a medical context’ (U = 12,704, Z = −3.6, *p* < 0.01) and ‘I know where to find reliable information (e.g., on evidence, general use) about AI in healthcare’ (U = 11,225, Z = −4.22, *p* < 0.01). The detailed subgroup analysis regarding sex, age and previous professional use of AI can be found in Supplementary Appendix 6. Further subgroup analyses regarding intensive care experience (in years), additional designation in intensive care medicine and leader ship position was conducted but findings were statistically not significant.

## 4 Discussion

### 4.1 Attitude toward AI in general and in healthcare

The present study found that physicians with intensive care experience in Germany tend to have a positive attitude toward AI in general and specifically in healthcare. This finding corroborates the conclusions of earlier studies conducted with physicians, from Germany (16), China (17) or healthcare workers (including physicians) from Bangladesh (18). In our study, the gender and age of the respondents had a negligible influence on their attitude. Investigating various groups of physicians, Heinrichs et al. arrived at conclusions that were analogous (16). Their study conducted found that demographic factors played only a minor role in shaping attitudes. A study undertaken in Bahrain by Al-Medfa et al. also yielded conclusions that were consistent with those previously mentioned (19). Nevertheless, this contrasts with findings from Bangladesh, where younger (18–35 years old) male participants demonstrated greater knowledge and a more positive attitude toward AI in healthcare (18).

However, a significant disparity was identified between physicians with prior experience of working with AI and those with no such experience. The proportion of the first group in our study (22.9%) corresponds to that of a study from China (22.8%), as does the finding that physicians who had already used AI in healthcare had a significantly more positive attitude toward it (17). In addition to the finding that experience with and use of AI lead to a more positive attitude, the extant literature also describes that concerns may decrease with increasing familiarity and professional use (16). Therefore, despite the low effect sizes observed in this case, this correlation should by no means be ignored. Further research should especially investigate the causal direction between the previous use and the attitude. Given our data it remains uncertain whether prior utilization of AI results in a more positive attitude, or whether a positive attitude leads to greater openness to AI and subsequent use. In this context, Heinrichs et al. found that both professional use of AI and the intention to use AI professionally were associated with higher enthusiasm and lower skepticism (16). Nevertheless, these findings do not totally resolve the issue of causality.

### 4.2 Requirements in the context of AI in healthcare

The two items “The results of AI applications must always be comprehensible to the treating physician” and “Objective values are not always sufficient for making medical decisions” received the highest approval ratings in this study, with scores of 4.25 and 4.36 respectively. These findings are consistent with the expert interviews previously conducted as part of the overarching research project (7) and point to two important implications for the design and application of AI in healthcare. First, many AI applications, particularly those employing neural networks, are based on black-box models. This means that the decisions made by the AI cannot necessarily be explained by the user. However, given the requirement for explainability, it is imperative that these systems receive particular consideration in future research. Second, since objective values are not sufficient for making medical decisions, implicit knowledge is necessary in decision-making. This type of knowledge is challenging to formalize and, as a result, can only be represented in AI systems to a limited extent. Given that the objective should not be to replace doctors (20) and that there are distinct tasks requiring empathy and human attention (21), it is essential that physicians continue to contribute their expertise to assess the decisions proposed by AI. Based on this evaluation, they should either accept or consciously reject these decisions.

### 4.3 Information on AI in healthcare

The survey identifies the potential for improvement in the availability of reliable information about AI in healthcare. Given a strong positive correlation between existing knowledge and positive attitudes towards AI in healthcare (18), it is concerning that only 7.5% totally agreed that they know where to find such information and 21.4% strongly disagreed. While literature describes attending AI conferences and learning through research articles/journals to be positively associated with good knowledge and positive attitudes (18), physicians in our survey considered communication about AI in the medical community (e.g., professional associations, conferences) to be rather inappropriate. Only 3.8% considered it as totally appropriate. Despite a positive and promising attitude of healthcare students, these future physicians showed few knowledge and limited skills in working with AI (22) reinforcing the need for structured communication and information on AI in healthcare.

### 4.4 General implications

The usefulness of AI is contingent on its integration into software or machines, as the benefits of the technology become apparent only in such contexts. The spectrum of potential applications is extensive. It includes domains such as medical imaging and diagnostic services, pandemic response, and virtual patient care. Other areas are enhanced patient retention and adherence to treatment plans, as well as the reduction of administrative burden on healthcare professionals. Further examples are the promotion of innovation in pharmaceuticals and vaccines, the monitoring of patient compliance with exercise regimens, and the use of gait analysis in technology-assisted rehabilitation (23).

Nevertheless, a reverse influence can also be assumed, whereby attitudes toward AI affect the acceptance of the corresponding software or machines. Therefore, the relatively positive attitude toward AI should be leveraged to identify meaningful application scenarios and use the technology to develop ways to improve healthcare and make it more efficient.

### 4.5 Limitations

We conducted a comprehensive questionnaire survey, contacting 7,475 physicians. Of these, 620 (8.3%) responded. A total of 445 returned questionnaires met the inclusion criteria and were subsequently analyzed. Despite contacting a large number of physicians and receiving a significant number of evaluable questionnaires, the major limitation of our study is the uneven distribution of participant characteristics compared to the overall physician population. These characteristics include gender, age and the proportion of physicians in leadership positions.

Regarding gender, 16.9% of the physicians in our study were female. According to data from the German Medical Association, the corresponding rates are 25.4% in surgery (including orthopedics), 41.9% in internal medicine and 44.1% in anesthesiology (24). In terms of age, a direct comparison is only approximate, as age categories were defined differently in our study. Focusing on anesthesiology, surgery (including orthopedics) and internal medicine, according to the German Medical Association, however, 16.7% of physicians are under 40 years old, 30.3% are aged 40–49, 28.5% are aged 50–59, and 24.5% are aged 60 or over (24).

Similarly, our survey clearly overrepresented physicians in leadership positions, whereas they account for between 7.7% and 16.6% for anesthesiology and surgery, respectively (24). This discrepancy can be explained by two factors relating to the address provider. First, we excluded orthopedics and trauma surgeons, as well as general and visceral surgeons, and internists working solely as ward physicians, since they do not necessarily have intensive care experience. Second, younger physicians often change departments or hospitals and are therefore frequently no longer available at their registered address. In contrast, older physicians in management positions tend to be more stable in this regard.

While our sample does not fully reflect the underlying physician population, the advantage is that the respondents represent key decision-makers regarding the integration of AI into intensive care.

A further point for discussion is that the Attari-12 tool is not specific to healthcare. On the one hand it is unclear to what extent the attitudes measured by the tool are applicable to healthcare. On the other hand, since the Attari-12 items were presented within a survey focused on healthcare, participants may have been primed by the medical context, potentially influencing their responses.

Importantly, when we asked participants to assess the statement “I am positive about the use of AI in a medical context”, we observed even higher levels of agreement than with the Attari-12. This indicates that general attitudes toward AI do not necessarily translate directly to attitudes in healthcare settings, highlighting the need to assess context-specific perspectives. By expanding the survey with additional items specifically targeting the use of AI in healthcare, we were able to gain relevant requirements and perspectives that are directly applicable to the medical domain.

Lastly, there is discussion about whether attitude toward AI is a uni- or bidimensional construct with positive and negative factors. Keeping this in mind one could question the use of the Attari-12 since it might risk missing ambivalence toward AI (25). Nevertheless, we deemed the tool eligible given two reasons: First, it provides a quick and efficient way of testing AI attitudes. Second, it represents three different facets (cognitive, affective, and behavioral) giving a broad assessment of the attitudes. Meanwhile, Gnambs et al. further developed the Attari-12 to a more economical scale, the Attari-WHE, focusing on three fields of application: Work, Healthcare, Education (26). Further investigations should consider using the newer tool when assessing attitudes toward AI.

In addition, it should be noted that multiple testing poses a potential problem in our study. Performing many statistical tests increases the likelihood of type 1 errors, i.e., the false rejection of the null hypothesis even though it is actually true.

### 4.6 Future research

In addition to the results presented in this article, our study highlights areas for future research. Two points are particularly relevant. First, younger and female physicians as well as physicians without leadership position were underrepresented in our study, so future studies should examine their attitudes to see if they are comparatively positive. Second, the causal relationship between conscious professional use of AI and a positive attitude (both in general and specifically in healthcare) remains to be clarified. Future (longitudinal) studies are needed to clarify whether AI use fosters a positive attitude or whether a pre-existing positive attitude drives AI use.

## 5 Conclusions

While the sample was predominantly composed of male and older physicians in leading positions, potentially limiting generalizability to younger or female physicians and those without leading positions, the results provide valuable insights into key decision-makers’ attitudes toward AI in intensive care settings. The main finding of our study is that physicians with intensive care experience have a relatively positive attitude toward AI in general and especially in healthcare. Nevertheless, some limitations assessed by physicians should be taken into account. First, the results of AI applications should always be comprehensible. Second, objective values alone do not suffice to make medical decisions. Third, there is a need to provide reliable information on AI in healthcare for physicians and to clearly communicate how physicians can further their education in this area. Finally, the positive attitude of physicians should be used to make healthcare systems better, more efficient, and more sustainable with the help of AI.

## Data Availability

Primary data of the survey are not publicly available due to data protection reasons. Requests for access should be directed to the corresponding author.
