# Supplementary figures and images for: Attitude and perception toward artificial intelligence among German physicians with intensive care experience: a survey study
Source: Front Health Serv. 2026 Feb 5;5:1721620. doi: 10.3389/frhs.2025.1721620 (PMC12916590; doi:10.3389/frhs.2025.1721620)

## Appendix 1. Excerpt of the questionnaire (p. 1, 4, 5, 14)


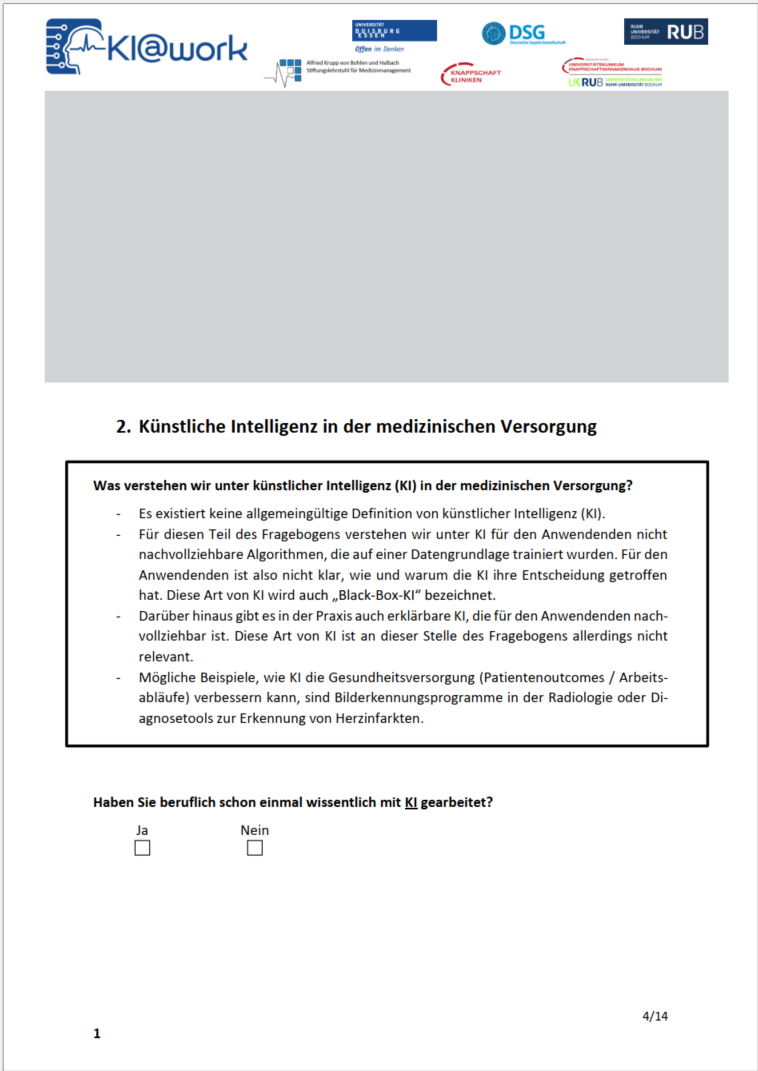

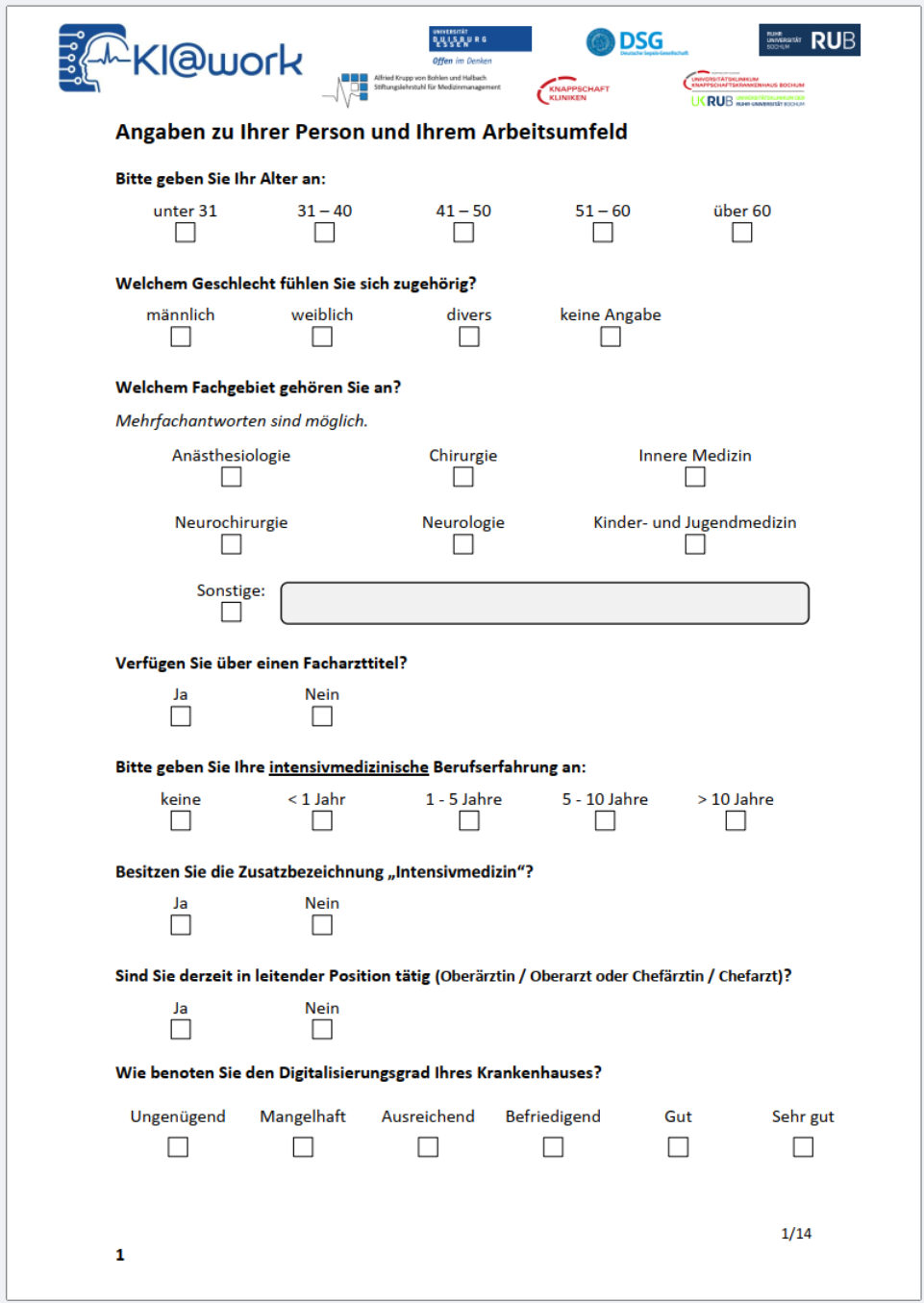

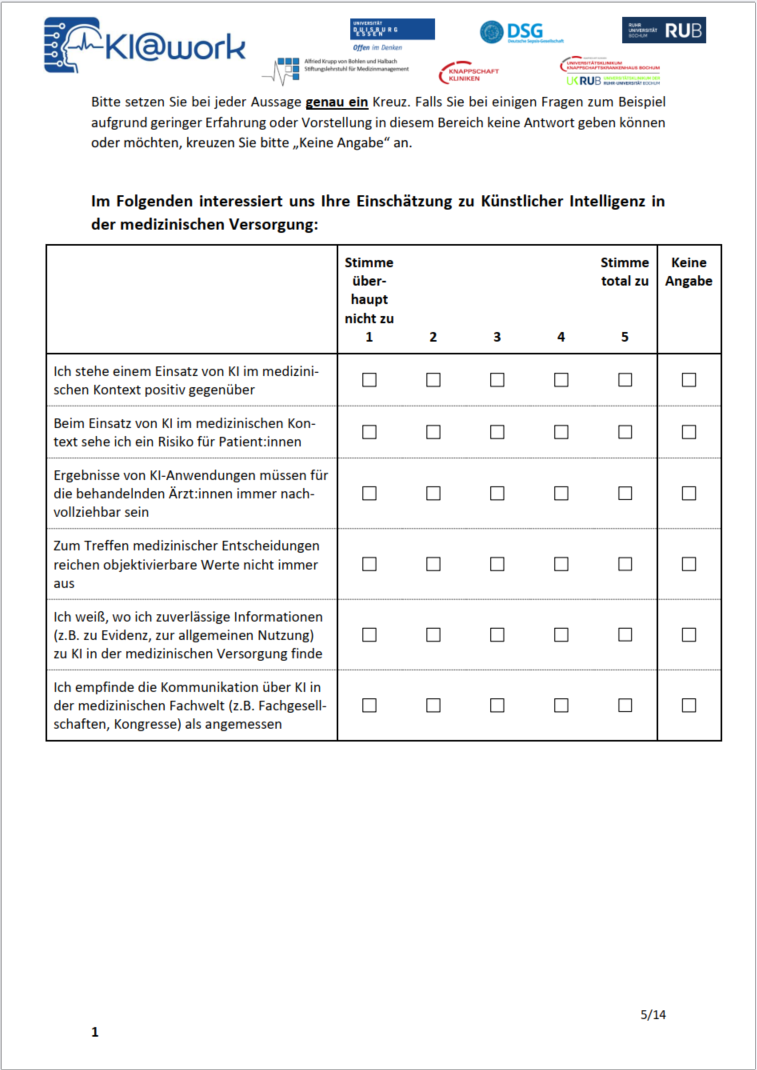

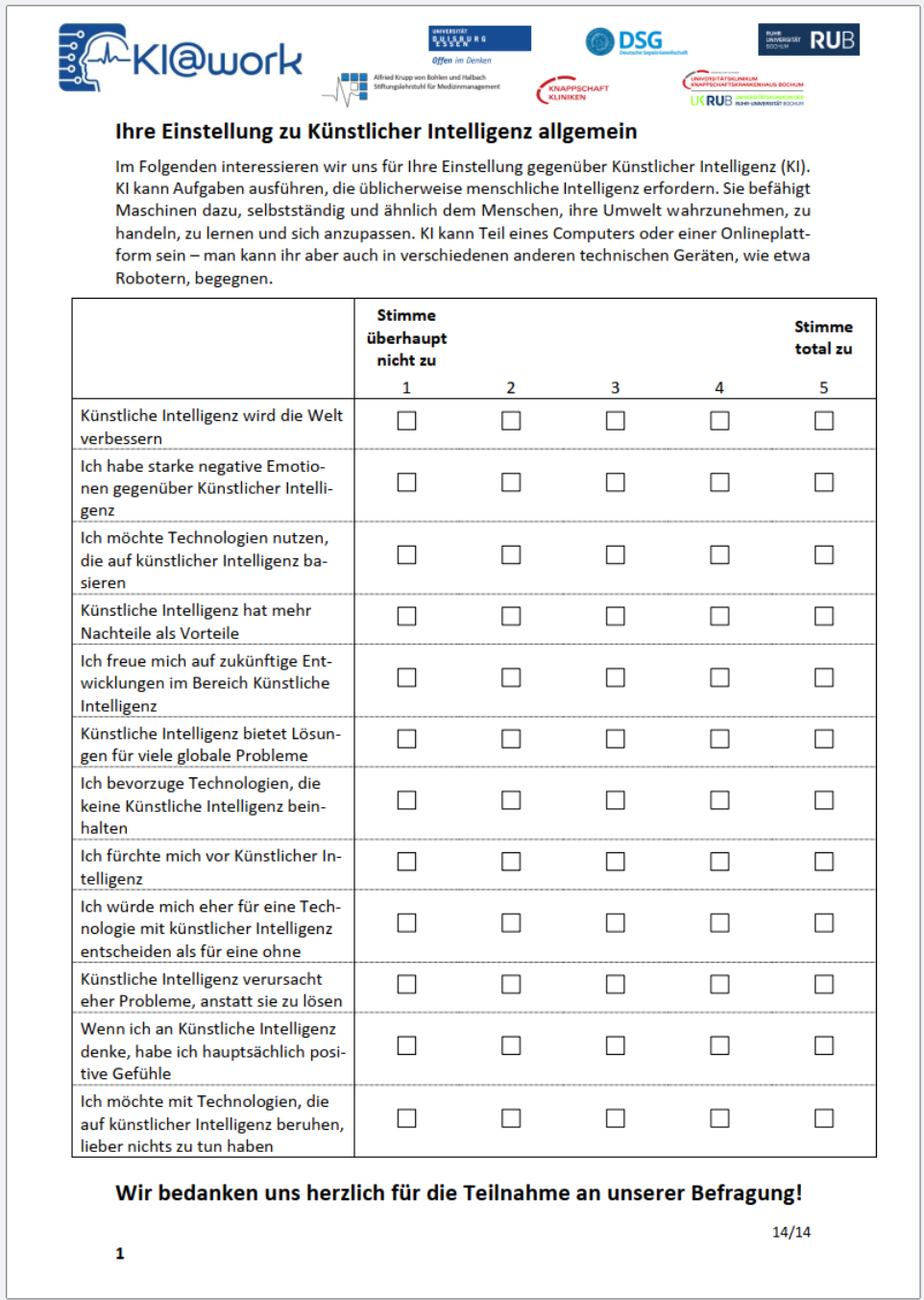

Supplement: Supplementary file 1 [file Table1.docx]
