# Supplementary material for: Attitude and perception toward artificial intelligence among German physicians with intensive care experience: a survey study
Source: Front Health Serv. 2026 Feb 5;5:1721620. doi: 10.3389/frhs.2025.1721620 (PMC12916590; doi:10.3389/frhs.2025.1721620)
Supplement: Supplementary file 2 [file Table2.docx]

## Appendix 2. Frequency and distribution of Attari-12 items

| **Item** | **1  (= strongly disagree)** | **2** | **3** | **4** | **5  (= totally agree)** |
| --- | --- | --- | --- | --- | --- |
| AI will make this world a better place. | 25 (5.6%) | 61 (13.7%) | 167 (37.5%) | 154 (34.6%) | 38 (8.5%) |
| I have strong negative emotions about AI. | 120 (27.0%) | 151 (33.9%) | 111 (24.9%) | 44 (9.9%) | 19 (4.3%) |
| I want to use technologies that rely on AI. | 8 (1.8%) | 25 (5.6%) | 102 (22.9%) | 200(44.9%) | 110 (24.7%) |
| AI has more disadvantages than advantages. | 72 (16.2%) | 151 (33.9%) | 148 (33.3%) | 60 (13.5%) | 14 (3.1%) |
| I look forward to future AI developments. | 11 (2.5%) | 35 (7.9%) | 92 (20.7%) | 191 (42.9%) | 116 (26.1%) |
| AI offers solutions to many world problems. | 24 (5.4%) | 59 (13.3%) | 142 (31.9%) | 164 (36.9%) | 56 (12.6%) |
| I prefer technologies that do not feature AI. | 128 (28.8%) | 146 (32.8%) | 103 (23.1%) | 48 (10.8%) | 20 (4.5%) |
| I am afraid of AI. | 195 (43.8%) | 122 (27.4%) | 76 (17.1%) | 42 (9.4%) | 10 (2.2%) |
| I would rather choose a technology with AI than one without it. | 28 (6.3%) | 70 (15.7%) | 188 (42.2%) | 118 (26.5%) | 41 (9.2%) |
| AI creates problems rather than solving them. | 87 (19.6%) | 170 (38.2%) | 146 (32.8%) | 30 (6.7%) | 12 (2.7%) |
| When I think about AI, I have mostly positive feelings. | 40 (9.0%) | 76 (17.1%) | 183 (41.1%) | 115 (25.8%) | 31 (7.0%) |
| I would rather avoid technologies that are based on AI. | 228 (51.2%) | 120 (27.0%) | 73 (16.4%) | 15 (3.4%) | 9 (2.0%) |
