# Supplementary material for: Attitude and perception toward artificial intelligence among German physicians with intensive care experience: a survey study
Source: Front Health Serv. 2026 Feb 5;5:1721620. doi: 10.3389/frhs.2025.1721620 (PMC12916590; doi:10.3389/frhs.2025.1721620)
Supplement: Supplementary file 4 [file Table4.docx]

## Appendix 4. Further subgroup analysis of Attari-12 items (Correlation/Association and effect size)

|  | **Additional designation in intensive care medicine*** | | **Intensive care experience**** | | **Leadership position*** | |
| --- | --- | --- | --- | --- | --- | --- |
|  | **p** | **effect size** | **p** | **effect size** | **p** | **effect size** |
| AI will make this world a better place. | n.s. | - | n.s. | - | n.s. | - |
| I have strong negative emotions about AI. | n.s. | - | n.s. | - | 0.01 | 0.01 |
| I want to use technologies that rely on AI. | n.s. | - | n.s. | - | n.s. | - |
| AI has more disadvantages than advantages. | n.s. | - | n.s. | - | n.s. | - |
| I look forward to future AI developments. | n.s. | - | n.s. | - | n.s. | - |
| AI offers solutions to many world problems. | n.s. | - | n.s. | - | n.s. | - |
| I prefer technologies that do not feature AI. | 0.035 | 0.01 | n.s. | - | n.s. | - |
| I am afraid of AI. | n.s. | - | n.s. | - | n.s. | - |
| I would rather choose a technology with AI than one without it. | n.s. | - | n.s. | - | n.s. | - |
| AI creates problems rather than solving them. | n.s. | - | n.s. | - | n.s. | - |
| When I think about AI, I have mostly positive feelings. | n.s. | - | n.s. | - | n.s. | - |
| I would rather avoid technologies that are based on AI. | n.s. | - | n.s. | - | n.s. | - |

** Mann-Whitney U Test with r^2^ effect size; **Kruskal-Wallis Test with Kendall’s Tau c; The level of statistical significance is set at* *α=0.05 (p ≤ 0.05).*
