# Supplementary material for: Attitude and perception toward artificial intelligence among German physicians with intensive care experience: a survey study
Source: Front Health Serv. 2026 Feb 5;5:1721620. doi: 10.3389/frhs.2025.1721620 (PMC12916590; doi:10.3389/frhs.2025.1721620)
Supplement: Supplementary file 5 [file Table5.docx]

## Appendix 5. Frequency and distribution of AI items

| **Item** | **Number of answers** | **1  (=strongly disagree)** | **2** | **3** | **4** | **5  (=totally agree)** |
| --- | --- | --- | --- | --- | --- | --- |
| I am positive about the use of AI in a medical context | 442 | 9 (2.0%) | 26 (5.9%) | 67 (15.2%) | 207 (46.8%) | 133 (30.1%) |
| I see a risk for patients when AI is used in a medical context | 441 | 31 (7.0%) | 155 (35.1%) | 146 (33.1%) | 91 (20.6%) | 18 (4.1%) |
| The results of AI applications must always be comprehensible to the treating physician | 442 | 9 (2.0%) | 20 (4.5%) | 28 (6.3%) | 102 (23.1%) | 283 (64.0%) |
| Objective values are not always sufficient for making medical decisions | 441 | 5 (1.1%) | 14 (3.2%) | 37 (8.4%) | 148 (33.6%) | 237 (53.7%) |
| I know where to find reliable information (e.g., on evidence, general use) about AI in healthcare | 426 | 91 (21.4%) | 133 (31.2%) | 89 (20.9%) | 81 (19.0%) | 32 (7.5%) |
| I consider communication about AI in the medical community (e.g., professional associations, conferences) to be appropriate | 422 | 42 (10.0%) | 140 (33.2%) | 149 (35.3%) | 75 (17.8%) | 16 (3.8%) |
