# Supplementary material for: Attitude and perception toward artificial intelligence among German physicians with intensive care experience: a survey study
Source: Front Health Serv. 2026 Feb 5;5:1721620. doi: 10.3389/frhs.2025.1721620 (PMC12916590; doi:10.3389/frhs.2025.1721620)
Supplement: Supplementary file 6 [file Table6.docx]

## Appendix 6. Correlation/association and effect sizes of AI items

|  | **sex*** | | **age groups**** | | **previous use of AI*** | |
| --- | --- | --- | --- | --- | --- | --- |
|  | **p** | **effect size** | **p** | **effect size** | **p** | **effect size** |
| I am positive about the use of AI in a medical context | n.s. | - | n.s. | - | < 0.001 | 0.03 |
| I see a risk for patients when AI is used in a medical context | n.s. | - | n.s. | - | n.s. | - |
| The results of AI applications must always be comprehensible to the treating physician | n.s. | - | n.s. | - | n.s. | - |
| Objective values are not always sufficient for making medical decisions | n.s. | - | n.s. | - | n.s. | - |
| I know where to find reliable information (e.g., on evidence, general use) about AI in healthcare | 0.003 | 0.02 | 0.007 | 0.069 | < 0.001 | 0.04 |
| I consider communication about AI in the medical community (e.g., professional associations, conferences) to be appropriate | n.s. | - | n.s. | - | n.s. | - |

** Mann-Whitney U Test with r^2^ effect size; **Kruskal-Wallis Test with Kendall’s Tau c; The level of statistical significance is set at* *α=0.05 (p ≤ 0.05).*
